# Supplementary material for: Dental distrust and discrimination: a nationally representative perspective on LGBTQ+ adults' experiences with oral health care
Source: Front Oral Health. 2026 Jul 9;7:1843339. doi: 10.3389/froh.2026.1843339 (PMC13417903; doi:10.3389/froh.2026.1843339)
Supplement: Supplementary file 1 [file Supplementaryfile1.docx]

**Appendix A: Multivariable Regression Models for Discrimination/EDSOC Scores by LGBTQ+ Status**

| **Variable** | **Beta** | **95% CI** | **p-value** |
| --- | --- | --- | --- |
| **LGBTQ+ Status** |  |  |  |
| Non-LGBTQ+ | ref | ref | ref |
| *LGBTQ+* | *0.16* | *0.11, 0.21* | *<0.001* |
| **Age in years** |  |  |  |
| 18-29 | ref | ref | ref |
| 30-44 | 0.00 | -0.06, 0.05 | >0.9 |
| *45-59* | *-0.20* | *-0.26, -0.15* | *<0.001* |
| *60+* | *-0.41* | *-0.46, -0.35* | *<0.001* |
| **Race/ethnicity** |  |  |  |
| White | ref | ref | ref |
| *Asian* | *0.35* | *0.26, 0.44* | *<0.001* |
| *Black* | *0.22* | *0.17, 0.27* | *<0.001* |
| *Hispanic* | *0.28* | *0.23, 0.33* | *<0.001* |
| *Other* | *0.26* | *0.16, 0.32* | *<0.001* |
| **Annual household income** |  |  |  |
| Less than $30,000 | ref | ref | ref |
| *$30,000 to under $60,000* | *-0.24* | *-0.29, -0.20* | *<0.001* |
| *$60,000 to under $100,000* | *-0.34* | *-0.38, -0.29* | *<0.001* |
| *$100,000 or more* | *-0.39* | *-0.43, -0.34* | *<0.001* |
| **Highest education level achieved** |  |  |  |
| Less than high school | ref | ref | ref |
| *High school graduate/equivalent* | *-0.22* | *-0.30, -0.13* | *<0.001* |
| *Some college/associate’s degree* | *-0.33* | *-0.41, -0.25* | *<0.001* |
| *Bachelor’s degree* | *-0.41* | *-0.49, -0.32* | *<0.001* |
| *Postgraduate/professional degree* | *-0.37* | *-0.46, -0.29* | *<0.001* |
| **Employment** |  |  |  |
| Working – paid or self-employed | ref | ref | ref |
| Not working – retired/disabled/other | 0.00 | -0.03, 0.04 | 0.8 |
| **Dental insurance** |  |  |  |
| Have dental insurance | ref | ref | ref |
| *Do not have dental insurance* | *0.16* | *0.13, 0.20* | *<0.001* |

Ref = reference category; models control for age in years, race/ethnicity, highest education level achieved, annual household income, employment status, and dental insurance status; italicized row = significant at the p<0.05 level; adjusted R^2^ = 0.12.

**Appendix B: Multivariable Regression Models for Discrimination/EDSOC Scores by Sexual Orientation**

| **Variable** | **Beta** | **95% CI** | **p-value** |
| --- | --- | --- | --- |
| **Sexual orientation** |  |  |  |
| Straight | ref | ref | ref |
| Bisexual | 0.04 | -0.04, .011 | 0.3 |
| *Gay/lesbian* | *0.13* | *0.05, 0.22* | *0.002* |
| *Other* | *0.15* | *0.07, 0.23* | *<0.001* |
| *Questioning* | *0.62* | *0.48, 0.77* | *<0.001* |
| **Age in years** |  |  |  |
| 18-29 | ref | ref | ref |
| 30-44 | -0.01 | -0.06, 0.05 | 0.8 |
| *45-59* | *-0.21* | *-0.27, -0.16* | *<0.001* |
| *60+* | *-0.41* | *-0.47, -0.35* | *<0.001* |
| **Race/ethnicity** |  |  |  |
| White | ref | ref | ref |
| *Asian* | *0.33* | *0.24, 0.43* | *<0.001* |
| *Black* | *0.22* | *0.17, 0.27* | *<0.001* |
| *Hispanic* | *0.27* | *0.22, 0.32* | *<0.001* |
| *Other* | *0.24* | *0.17, 0.30* | *<0.001* |
| **Annual household income** |  |  |  |
| Less than $30,000 | ref | ref | ref |
| *$30,000 to under $60,000* | *-0.24* | *-0.29, -0.20* | *<0.001* |
| *$60,000 to under $100,000* | *-0.33* | *-0.38, -0.29* | *<0.001* |
| *$100,000 or more* | *-0.38* | *-0.43, -0.33* | *<0.001* |
| **Highest education level achieved** |  |  |  |
| Less than high school | ref | ref | ref |
| *High school graduate/equivalent* | *-0.22* | *-0.30, -0.14* | *<0.001* |
| *Some college/associate’s degree* | *-0.34* | *-0.42, -0.26* | *<0.001* |
| *Bachelor’s degree* | *-0.42* | *-0.50, -0.33* | *<0.001* |
| *Postgraduate/professional degree* | *-0.38* | *-0.47, -0.30* | *<0.001* |
| **Employment** |  |  |  |
| Working – paid or self-employed | ref | ref | ref |
| Not working – retired/disabled/other | 0.00 | -0.04, 0.03 | >0.9 |
| **Dental insurance** |  |  |  |
| Have dental insurance | ref | ref | ref |
| *Do not have dental insurance* | *0.16* | *0.13, 0.20* | *<0.001* |

Ref = reference category; models control for age in years, race/ethnicity, highest education level achieved, annual household income, employment status, and dental insurance status; italicized row = significant at the p<0.05 level; adjusted R^2^ = 0.16.

**Appendix C: Multivariable Regression Models for Discrimination/EDSOC Scores by Gender Identity**

| **Variable** | **Beta** | **95% CI** | **p-value** |
| --- | --- | --- | --- |
| **Gender identity** |  |  |  |
| Male | ref | ref | ref |
| Female | -0.02 | -0.05, 0.01 | 0.2 |
| *Other* | *0.64* | *0.45, 0.84* | *<0.001* |
| Transgender | 0.24 | -0.07, 0.55 | 0.13 |
| **Age in years** |  |  |  |
| 18-29 | ref | ref | ref |
| 30-44 | -0.02 | -0.08, 0.03 | 0.4 |
| *45-59* | *-0.23* | *-0.28, -0.17* | *<0.001* |
| *60+* | *-0.42* | *-0.48, -0.37* | *<0.001* |
| **Race/ethnicity** |  |  |  |
| White | ref | ref | ref |
| *Asian* | *0.33* | *0.24, 0.43* | *<0.001* |
| *Black* | *0.22* | *0.17, 0.27* | *<0.001* |
| *Hispanic* | *0.27* | *0.22, 0.32* | *<0.001* |
| *Other* | *0.25* | *0.19, 0.31* | *<0.001* |
| **Annual household income** |  |  |  |
| Less than $30,000 | ref | ref | ref |
| *$30,000 to under $60,000* | *-0.23* | *-0.27, -0.18* | *<0.001* |
| *$60,000 to under $100,000* | *-0.33* | *-0.38, -0.28* | *<0.001* |
| *$100,000 or more* | *-0.39* | *-0.44, -0.34* | *<0.001* |
| **Highest education level achieved** |  |  |  |
| Less than high school | ref | ref | ref |
| *High school graduate/equivalent* | *-0.21* | *-0.29, -0.13* | *<0.001* |
| *Some college/associate’s degree* | *-0.32* | *-0.40, -0.24* | *<0.001* |
| *Bachelor’s degree* | *-0.40* | *-0.48, -0.31* | *<0.001* |
| *Postgraduate/professional degree* | *-0.36* | *-0.45, -0.28* | *<0.001* |
| **Employment** |  |  |  |
| Working – paid or self-employed | ref | ref | ref |
| Not working – retired/disabled/other | 0.00 | -0.03, 0.04 | 0.8 |
| **Dental insurance** |  |  |  |
| Have dental insurance | ref | ref | ref |
| *Do not have dental insurance* | *0.16* | *0.12, 0.19* | *<0.001* |

Ref = reference category; models control for age in years, race/ethnicity, highest education level achieved, annual household income, employment status, and dental insurance status; italicized row = significant at the p<0.05 level; adjusted R^2^ = 0.12.

**Appendix D: Multivariable Regression Models for Microaggression/DOCS Scores by LGBTQ+ Status**

| **Variable** | **Beta** | **95% CI** | **p-value** |
| --- | --- | --- | --- |
| **LGBTQ+ Status** |  |  |  |
| Non-LGBTQ+ | ref | ref | ref |
| *LGBTQ+* | *0.08* | *0.03, 0.13* | *0.003* |
| **Age in years** |  |  |  |
| 18-29 | ref | ref | ref |
| 30-44 | 0.01 | -0.06, 0.07 | 0.9 |
| *45-59* | *-0.12* | *-0.19, -0.06* | *<0.001* |
| *60+* | *-0.32* | *-0.38, -0.26* | *<0.001* |
| **Race/ethnicity** |  |  |  |
| White | ref | ref | ref |
| *Asian* | *0.23* | *0.13, 0.33* | *<0.001* |
| *Black* | *0.16* | *0.11, 0.22* | *<0.001* |
| *Hispanic* | *0.15* | *0.09, 0.20* | *<0.001* |
| *Other* | *0.16* | *0.09, 0.23* | *<0.001* |
| **Annual household income** |  |  |  |
| Less than $30,000 | ref | ref | ref |
| *$30,000 to under $60,000* | *-0.15* | *-0.20, -0.10* | *<0.001* |
| *$60,000 to under $100,000* | *-0.27* | *-0.32, -0.22* | *<0.001* |
| *$100,000 or more* | *-0.33* | *-0.39, -0.28* | *<0.001* |
| **Highest education level achieved** |  |  |  |
| Less than high school | ref | ref | ref |
| *High school graduate/equivalent* | *-0.20* | *-0.29, -0.10* | *<0.001* |
| *Some college/associate’s degree* | *-0.29* | *-0.38, -0.20* | *<0.001* |
| *Bachelor’s degree* | *-0.39* | *-0.49, -0.30* | *<0.001* |
| *Postgraduate/professional degree* | *-0.44* | *-0.54, -0.34* | *<0.001* |
| **Employment** |  |  |  |
| Working – paid or self-employed | ref | ref | ref |
| Not working – retired/disabled/other | 0.03 | -0.01, -0.07 | 0.11 |
| **Dental insurance** |  |  |  |
| Have dental insurance | ref | ref | ref |
| *Do not have dental insurance* | *0.17* | *0.13, 0.21* | *<0.001* |

Ref = reference category; models control for age in years, race/ethnicity, highest education level achieved, annual household income, employment status, and dental insurance status; italicized row = significant at the p<0.05 level; adjusted R^2^ = 0.08.

**Appendix E: Multivariable Regression Models for Microaggression/DOCS Scores by Sexual Orientation**

| **Variable** | **Beta** | **95% CI** | **p-value** |
| --- | --- | --- | --- |
| **Sexual orientation** |  |  |  |
| Straight | ref | ref | ref |
| Bisexual | 0.07 | -0.01, 0.15 | 0.10 |
| Gay/lesbian | 0.03 | -0.07, 0.12 | 0.6 |
| Other | 0.09 | 0.00, 0.18 | 0.055 |
| *Questioning* | *0.29* | *0.13, 0.46* | *<0.001* |
| **Age in years** |  |  |  |
| 18-29 | ref | ref | ref |
| 30-44 | 0.00 | -0.06, 0.07 | >0.9 |
| *45-59* | *-0.13* | *-0.19, -0.06* | *<0.001* |
| *60+* | *-0.32* | *-0.39, -0.26* | *<0.001* |
| **Race/ethnicity** |  |  |  |
| White | ref | ref | ref |
| *Asian* | *0.23* | *0.13, 0.33* | *<0.001* |
| *Black* | *0.16* | *0.10, 0.21* | *<0.001* |
| *Hispanic* | *0.15* | *0.09, 0.20* | *<0.001* |
| *Other* | *0.16* | *0.09, 0.23* | *<0.001* |
| **Annual household income** |  |  |  |
| Less than $30,000 | ref | ref | ref |
| *$30,000 to under $60,000* | *-0.15* | *-0.20, -0.10* | *<0.001* |
| *$60,000 to under $100,000* | *-0.27* | *-0.32, -0.21* | *<0.001* |
| *$100,000 or more* | *-0.33* | *-0.39, -0.28* | *<0.001* |
| **Highest education level achieved** |  |  |  |
| Less than high school | ref | ref | ref |
| *High school graduate/equivalent* | *-0.21* | *-0.30, -0.11* | *<0.001* |
| *Some college/associate’s degree* | *-0.30* | *-0.39, -0.21* | *<0.001* |
| *Bachelor’s degree* | *-0.40* | *-0.50, -0.31* | *<0.001* |
| *Postgraduate/professional degree* | *-0.46* | *-0.55, -0.36* | *<0.001* |
| **Employment** |  |  |  |
| Working – paid or self-employed | ref | ref | ref |
| Not working – retired/disabled/other | 0.03 | -0.01, 0.07 | 0.12 |
| **Dental insurance** |  |  |  |
| Have dental insurance | ref | ref | ref |
| *Do not have dental insurance* | *0.16* | *0.12, 0.20* | *<0.001* |

Ref = reference category; models control for age in years, race/ethnicity, highest education level achieved, annual household income, employment status, and dental insurance status; italicized row = significant at the p<0.05 level; adjusted R^2^ = 0.08.

**Appendix F: Multivariable Regression Models for Microaggression/DOCS Scores by Gender Identity**

| **Variable** | **Beta** | **95% CI** | **p-value** |
| --- | --- | --- | --- |
| **Gender identity** |  |  |  |
| Male | ref | ref | ref |
| *Female* | *-0.12* | *-0.15, -0.08* | *<0.001* |
| *Other* | *0.42* | *0.18, 0.66* | *<0.001* |
| Transgender | 0.32 | -0.04, -0.68 | 0.082 |
| **Age in years** |  |  |  |
| 18-29 | ref | ref | ref |
| 30-44 | 0.00 | -0.06, 0.06 | >0.9 |
| *45-59* | *-0.13* | *-0.19, -0.06* | *<0.001* |
| *60+* | *-0.33* | *-0.39, -0.27* | *<0.001* |
| **Race/ethnicity** |  |  |  |
| White | ref | ref | ref |
| *Asian* | *0.21* | *0.11, 0.31* | *<0.001* |
| *Black* | *0.17* | *0.11, 0.23* | *<0.001* |
| *Hispanic* | *0.14* | *0.09, 0.20* | *<0.001* |
| *Other* | *0.16* | *0.09, 0.23* | *<0.001* |
| **Annual household income** |  |  |  |
| Less than $30,000 | ref | ref | ref |
| *$30,000 to under $60,000* | *-0.15* | *-0.20, -0.10* | *<0.001* |
| *$60,000 to under $100,000* | *-0.28* | *-0.33, -0.23* | *<0.001* |
| *$100,000 or more* | *-0.35* | *-0.41, -0.30* | *<0.001* |
| **Highest education level achieved** |  |  |  |
| Less than high school | ref | ref | ref |
| *High school graduate/equivalent* | *-0.20* | *-0.30, -0.11* | *<0.001* |
| *Some college/associate’s degree* | *-0.28* | *-0.37, -0.19* | *<0.001* |
| *Bachelor’s degree* | *-0.39* | *-0.48, -0.29* | *<0.001* |
| *Postgraduate/professional degree* | *-0.43* | *-0.53, -0.33* | *<0.001* |
| **Employment** |  |  |  |
| Working – paid or self-employed | ref | ref | ref |
| Not working – retired/disabled/other | 0.03 | 0.00, 0.07 | 0.079 |
| **Dental insurance** |  |  |  |
| Have dental insurance | ref | ref | ref |
| *Do not have dental insurance* | *0.16* | *0.12, 0.20* | *<0.001* |

Ref = reference category; models control for age in years, race/ethnicity, highest education level achieved, annual household income, employment status, and dental insurance status; italicized row = significant at the p<0.05 level; adjusted R^2^ = 0.09.
